# Quantitative Sensory Testing Across Chronic Pain Conditions and Use in Special Populations

**DOI:** 10.3389/fpain.2021.779068

**Published:** 2022-01-28

**Authors:** Kristen R. Weaver, Mari A. Griffioen, N. Jennifer Klinedinst, Elizabeth Galik, Ana C. Duarte, Luana Colloca, Barbara Resnick, Susan G. Dorsey, Cynthia L. Renn

**Affiliations:** ^1^Department of Pain and Translational Symptom Science, School of Nursing, University of Maryland, Baltimore, MD, United States; ^2^Center to Advance Chronic Pain Research (CACPR), University of Maryland, Baltimore, MD, United States; ^3^College of Health Sciences, School of Nursing, University of Delaware, Newark, DE, United States; ^4^Department of Organizational Systems and Adult Health, School of Nursing, University of Maryland, Baltimore, MD, United States; ^5^Department of Family and Community Health, School of Nursing, University of Maryland, Baltimore, MD, United States

**Keywords:** low back pain, temporomandibular joint disorder (TMD), irritable bowel syndrome (IBS), placebo, dementia, osteoarthritis

## Abstract

Chronic pain imposes a significant burden to the healthcare system and adversely affects patients' quality of life. Traditional subjective assessments, however, do not adequately capture the complex phenomenon of pain, which is influenced by a multitude of factors including environmental, developmental, genetic, and psychological. Quantitative sensory testing (QST), established as a protocol to examine thermal and mechanical sensory function, offers insight on potential mechanisms contributing to an individual's experience of pain, by assessing their perceived response to standardized delivery of stimuli. Although the use of QST as a research methodology has been described in the literature in reference to specific pain populations, this manuscript details application of QST across a variety of chronic pain conditions. Specific conditions include lower extremity chronic pain, knee osteoarthritis, chronic low back pain, temporomandibular joint disorder, and irritable bowel syndrome. Furthermore, we describe the use of QST in placebo/nocebo research, and discuss the use of QST in vulnerable populations such as those with dementia. We illustrate how the evaluation of peripheral sensory nerve function holds clinical promise in targeting interventions, and how using QST can enhance patient education regarding prognostic outcomes with particular treatments. Incorporation of QST methodology in research investigations may facilitate the identification of common mechanisms underlying chronic pain conditions, guide the development of non-pharmacological behavioral interventions to reduce pain and pain-related morbidity, and enhance our efforts toward reducing the burden of chronic pain.

## Introduction

Pain is a complex phenomenon where the intensity, characteristics, extent, and duration varies from person to person, and reflects the complex biopsychosocial interactions between genetic, developmental, environmental, and psychological factors (1). While many studies rely on Patient-Reported Outcomes Measures (PROM) reports of pain such as the 11-point Numeric Rating Scale (a unidimensional measure of the magnitude of pain), or the Short-Form McGill Pain Questionnaire (measure of pain characteristics), adding measures that include a person's perceived response to a standardized somatosensory testing protocol offers an opportunity to further characterize the pain experience (2). Importantly, the ability to implement personalized treatment plans necessitates the careful identification of individual pain characteristics (3). Quantitative sensory testing (QST) provides a complementary assessment of an individual's response to stimuli and offers information about the potential underlying mechanisms contributing to pain. Thus, incorporating QST may help to address recognized challenges in pain assessment across patient populations, and guide the development of non-pharmacological interventions to reduce pain, as detailed below.

QST was established by the German Research Network on Neuropathic Pain as a protocol to examine thermal and mechanical sensory function (4). It refers to the use of a set of standardized testing procedures that allows the examiner to measure and quantify somatosensory function in large (Aβ-fibers) and small sensory nerve fibers (Aδ- and C-fibers) with an aim to detect sensory loss (i.e., hypoesthesia, hypoalgesia) or sensory gain (i.e., hyperesthesia, hyperalgesia, allodynia) (4) (see Table 1). Two key components of chronic pain are peripheral and central sensitization. Peripheral sensitization results from increased excitability of nociceptors, leading to the development of hyperalgesia (14). Using QST, peripheral sensitization is reflected as a decrease in thermal (hot and cold) pain thresholds (6). Central sensitization is a result of increased excitability of neurons in the central nervous system (CNS), which leads to the development of allodynia in the immediate area of pain, as well as hyperalgesia and allodynia in surrounding regions (6). Central sensitization is identified using the QST measure of temporal summation, where applying rapidly repeated mechanical or thermal stimuli results in increased pain perception. Of note, central sensitization as measured by QST is distinct from central sensitization-related symptoms and diagnoses, as measured by the Central Sensitization Inventory, or CSI (15).

**Table 1:** Specific tests from the German Research Network on Neuropathic Pain QST protocol by chronic pain conditions of interest and Conditioned Pain Modulation.

**Sensory function quantified and modality**	**Equipment**	**Chronic pain condition that used test**	**Neuroplasticity system tested**	**Reported sensory abnormality**
Thermal detection threshold Cold/Warm^a^ Paradoxical heat sensation^a^	Pathway thermal stimulator^b^	LEFx chronic pain TMD chronic pain KOA chronic pain	Peripheral	LEFx chronic pain—decreased warmth detection threshold (5)
		LEFx chronic pain TMD chronic pain	Central	
Thermal pain threshold Cold/Heat^a^	Pathway thermal stimulator^b^	LEFx chronic pain TMD chronic pain KOA chronic pain Chronic LBP	Peripheral	Chronic LBP—decreased heat pain threshold (6)
Mechanical detection threshold Touch^a^	Touch test sensory evaluation kit	LEFx chronic pain	Peripheral	
Wind-up ratio Mechanical temporal Summation^a^	Pinprick stimulator^e^	KOA chronic pain		KOA chronic pain—enhanced TS (7–9)
Vibration detection^a^	Graduated tuning fork^c^	LEFx chronic pain	Peripheral	
Pressure pain threshold^a^	Pressure algometer^b^	LEFx chronic pain KOA chronic pain Chronic LBP	Peripheral	KOA chronic pain—lower PPT Chronic LBP—lower PPT (10)
Mechanical pain threshold^f^ Pin prick stimuli^a^	Pinprick stimulator^d^			
Stimulus response function^f^ Mechanical pain sensitivity Pin prick stimuli Dynamic mechanical allodynia Tactile stimulators	Pinprick stimulator^e^			
Conditioned Pain Modulation	Test stimulus: electrical, heat, rectal distention Conditioning stimulus: cold pressor, hot pressor, cold pain, heat stimulus	IBS chronic pain Chronic LBP	Central	Diminished CPM in patients with IBS (11, 12) Decreased descending inhibitory control in patients with LBP (13)

a *Rolke et al. (4)*,

b *Medoc; Ramat, Israel*,

c *Rydel-Seiffer, US Neurologicals*,

d *Owen Mumford, Marietta, GA, USA*,

e *MRC Systems GmbH, Heidelberg, Germany*,

f *Investigators are not always able to include all tests in their protocol, but have to prioritize those most salient to their patient population and research question*.

*LEFx, Lower extremity fracture; TMD, temporomandibular disorder; KOA, knee osteoarthritis; LBP, low back pain; IBS, irritable bowel syndrome*.

Another component of chronic pain is a decrease in descending inhibitory control in the CNS. Changes in central inhibitory pain modulation can be identified by using the conditioned pain modulation test (13). CPM testing involves applying a noxious stimulus (thermal or mechanical) to a region remote from the painful site. If descending inhibitory control is functioning properly, the initial source of pain will be less intense during the application of the remote stimulus (16). QST measures reported pain based on standardized screens or via physical means to measure pain thresholds and accommodation to varying levels of stimuli, and can be used to better predict pain treatments and outcomes (17). For instance, QST has successfully been used to reveal decreased sensitivity to stimuli in persons with lower back pain and lower extremity fractures (5, 18) and increased sensitivity to stimuli in persons with knee osteoarthritis (19). Furthermore, QST can aid in characterizing peripheral sensory nerve function, which can then be correlated with a wide range of other factors such as genetic biomarkers, treatment efficacy, and biobehavioral activities to generate risk profiles and guide treatment. QST has also been shown to predict analgesic effects (20).

The objective of this manuscript is to review the application of tests from the QST protocol established by the German Research Network on Neuropathic Pain (DNFS) (4) across a variety of chronic pain conditions in our Omics Associated with Self-Management Interventions for Symptoms (OASIS) Center. Specific conditions include lower extremity (LE) chronic pain, knee osteoarthritis (KOA), chronic low back pain (LBP), temporomandibular joint disorder (TMD), and irritable bowel syndrome (IBS). Furthermore, we describe the use of QST in placebo/nocebo research, and discuss the use of QST in vulnerable populations such as those with Alzheimer's disease (AD). Relevant literature for each condition was retrieved by the co-author whose research surrounds that population of interest. As chronic pain imposes significant burden to the healthcare system and adversely affects patients' quality of life, utilizing QST methodology may offer insight on an individual's experience of pain, identify commonalities across chronic pain conditions, and direct tailored interventions, including non-pharmacological behavioral treatments, to reduce pain and pain-related morbidity.

## Lower Extremity Chronic Pain/Knee Osteoarthritis

The etiology of lower extremity (LE) chronic pain is wide-ranging (i.e. arthritis, injuries, surgeries) and contributes significantly to negative physical, psychological, social, emotional, and economical consequences for adults across the life-span (1). While the biological mechanism associated with LE chronic pain is poorly understood, changes in peripheral sensory nerve function assessed with QST have been identified in persons with chronic pain following lower extremity fractures and arthritis. For instance, hypoesthesia, decreased warmth detection threshold, has been noted in persons with LE-fracture related chronic pain (5). This mirrors what has been reported in persons with traumatic partial nerve injuries (21) and may be due to peripheral nerve injuries that have not fully healed (22). Changes in LE sensation (23), whether hypoesthesia or hyperesthesia, is important information as poor peripheral sensory nerve function is associated with decreased LE function and mobility limitations, especially in older adults (24). Furthermore, LE chronic pain is associated with an increased fall risk and falls have catastrophic consequences with increased morbidity and mortality (25). Therefore, evaluating peripheral sensory nerve function in LE chronic pain has the potential for clinicians to target interventions on mobility and balance that may be affected by these changes.

One cause of LE chronic pain, knee osteoarthritis (KOA), affects approximately one third of adults in the United States (26, 27). Pain is the most prevalent and troublesome symptom of KOA (28) leading patients to seek medical interventions for relief. However, KOA pain type and intensity often does not correlate to visual damage noted by radiography (29). KOA is a degenerative locally inflammatory disease in which both peripheral and central sensitization contribute to pain (30, 31). KOA pain symptoms are typically mild and nociceptive to start and triggered by weight bearing or physical activity. KOA induces tissue injury and/or inflammation leading to peripheral sensitization. Chronic KOA pain is multifactorial and qualitative work has described two types of pain experience: a constant aching and intermittent severe pain (28). Moreover, many adults with KOA experience alterations in endogenous pain inhibitory capacity that have neurobiologic and central mechanisms. Those adults with centralized KOA pain tend to be resistant to traditional pain treatment and have substantial pain even after knee replacement surgery (32). Therefore, phenotyping KOA pain is imperative for personalized treatment.

Quantitative sensory testing has merit for phenotyping KOA pain. Most studies in KOA use pressure pain thresholds at the affected joint and often a distal site to quantify pain sensitization (7, 33). Heat and cold pain detection and threshold have also been used but less often. In addition, temporal summation (TS) is often assessed, but Moore et al. found only weak correlations between TS and clinical pain ratings (7). This may be due to racial differences as one study found TS associated with clinical pain scores among non-Hispanic White people but not among Black people (8). It may also be due to variation of specific phenotypes of KOA pain where women and Black people with KOA were more likely to ascribe to high TS (9).

Pressure pain thresholds (PPT) are the most reliable measure of peripheral sensitization with consistent test-retest correlations and are able to differentiate KOA from healthy controls (33–36). Wylde et al. (36) tested PPT at the affected knee, contralateral knee, and forearm sites of adults with KOA one week apart and report intraclass correlation coefficients (ICC) for heat ranging from 0.52 to 0.70 and cold detection of 0.31–0.70 and for PPT 0.77–0.86. PPT are also correlated with the manual tender point count clinical measure, an assessment of peripheral sensitization (7). Consistently, adults with KOA have lower pressure pain thresholds at the affected joint than healthy controls (33, 37). Similar to PPT, TS is associated with pain severity but not severity of radiographic evidence of KOA (37).

QST signs of central pain mechanisms in KOA have been defined as low PPT and/or enhanced TS and/or allodynia (19), whereas localized pain at the joint but not at distal sites reflect more peripheral pain mechanisms. Using these QST tools together, testing pain sensitivity in adults with KOA at both the affected knee and a distal site (ipsilateral non-painful hand) resulted in the identification of five distinct pain phenotypes: #1—low pressure pain threshold, #2—average pain sensitivity across most modalities (pressure pain, heat and cold pain, temporal summation heat/cold), #3—high temporal summation of punctate pain, #4—high cold pain sensitivity, and #5—high heat pain sensitivity/high temporal summation of heat pain (9). These findings reflect the influence of distinct central and peripheral mechanisms on KOA pain. For example, those who are sensitive to heat pain have more pain after total knee surgery and consume more morphine post-surgery. Additionally, Carlesso et al. (38) identified pain susceptibility phenotypes of adults with KOA but free of pain and found that those with low PPT and high TS had twice the odds (OR 1.98) of developing persistent knee pain.

Understanding the mechanisms of KOA pain type and sensitivity using QST can facilitate the development of targeted treatment options and better patient education as to prognostic outcomes with particular treatments. For example, presence of TS was predictive of poor response to pain treatment with non-steroidal anti-inflammatory (NSAIDS) drugs (39). Of those adults with KOA who undergo costly knee replacement surgery, up to 44% will continue to have chronic knee pain months and even years post-operatively (32, 40). QST testing can help predict surgical outcomes as patients with neuropathic-type pain and widespread hyperalgesia, as indicated by widespread pressure and cold hyperalgesia on QST, are reported to experience persistent pain a year after total knee arthroplasty (41). Therefore, incorporating QST methodology in patient assessment can help detail mechanisms that underlie their experience of pain, offer prognostic indicators, and help guide the development of individualized treatment plans.

## Chronic Low Back Pain

Chronic low back pain (LBP) is among the most frequently diagnosed, most expensive to treat, and most debilitating pain condition in the United States (42, 43). It is estimated that approximately 40% of acute LBP patients will report having chronic LBP at 24 weeks after medical treatment for an acute episode of LBP (42). Furthermore, up to 90% of LBP patients have no identifiable etiology for their pain (44, 45). Being able to predict who will transition from an episode of acute LBP to developing chronic LBP will increase our ability to implement more aggressive therapies early in the treatment of the acute pain to prevent the transition to the chronic pain state. While many pain-related questionnaires provide insight into the patient's perception of LBP, unfortunately, the neurophysiological mechanisms underlying the development of chronic LBP remain unclear. The addition of QST measures to the assessment of LBP can provide valuable neurophysiological information that complements questionnaire data.

Research shows that patients with persistent LBP have evidence of peripheral and central sensitization, which may be key factors in their risk for chronic LBP (46, 47), and can be identified using the QST measures of thermal threshold, pressure pain threshold, temporal summation, as well as the conditioned pain modulation test. Changes in the QST measures during the acute LBP phase could be indicative of the potential for the acute LBP to transition into a chronic LBP state. Studies found peripheral sensitization was reflected as a decrease in the heat pain threshold (6), and pressure pain threshold (10). In patients with LBP (46, 47), central sensitization can be identified using temporal summation by applying rapidly repeated mechanical stimuli on the painful region resulting in increased pain perception. Conditioned pain modulation can be used to detect a decrease in CNS descending inhibitory control, which is another contributor to persistent LBP (13). Therefore, sensory alterations and increased pain sensitivity found in LBP patients can be measured using QST. Adding QST to clinical exams of LBP patients will allow clinicians to identify sensory function changes leading to increased pain, which will help enhance our understanding of the physiological mechanisms underlying this chronic pain condition.

## Temporomandibular Disorders

There are numerous types of chronic orofacial pain conditions including temporomandibular disorders (TMD) (48), also referred to as temporomandibular joint disorders or TMJ. TMJ is the abbreviation for the joint itself, while TMD are the actual conditions and their accompanying pain and inflammation. TMD affects approximately 5–12% of the general population (49–51) and are associated with significant personal and societal burdens (48). QST is used in various ways to determine underlying causes and potential methods for analgesia in these conditions, and has been used in measurement of various aspects of TMD-related pain (52–55).

In a large prospective cohort study (Orofacial Pain: Prospective Evaluation and Risk Assessment, OPPERA), QST was used as a predictor to identify onset of TMD in individuals who were otherwise healthy when enrolled in the trial (56). Risk factors in four main areas were identified. Psychosocial factors examined concurrent and pre-existing mental health issues. Age, sex, and race were among those identified as sociodemographic factors. Standard instruments that delivered painful stimuli were used to measure elevated response to pain. Genetic factors included both psychological traits and pain sensitivity (56). Baseline QST measurements were taken for pressure pain thresholds (PPT), mechanical pain stimuli, and thermal heat pain stimuli. Additionally, self-report information was gathered using questionnaires, and blood draws were performed (53). Increased incidence of TMD was found to be associated with the psychosocial risk factors of anxiety, depression, perceived stress levels, and sensitivity to somatic issues. Likewise, incident TMD was found to occur in those with greater temporal summation of heat pain and clinical factors such as autonomic measurements including heart rate at rest. Sociodemographic factors of being younger, woman, and White, as well as genetic factors, are associated with increased incidence and development or worsening of chronic TMD (53, 56).

Greenspan and colleagues also used QST to measure sensitivity to three modalities of nociception in TMD patients: blunt pressure pain, mechanical pinprick pain, and thermal heat pain (54). These investigators reported pain sensitivity to vary according to the total number of pain conditions a participant with TMD experienced, suggesting that the combination of pain conditions influences each nociceptive modality and QST-related measurements (54). Recently, we assessed thermal heat pain threshold and thermal heat pain tolerance in TMD participants as a proxy for the QST procedure. We found that Afro-American Blacks had lower pain tolerance in both TMD participants and healthy control participants (57). Additionally, we found women in the TMD cohort to have lower levels of both thermal heat pain threshold and thermal heat pain tolerance than men (58). Both results are in line with the literature on racial and sex effects with regard to pain sensitivity, as in experimental pain studies, women tend to be more sensitive to pain than men in measures of heat, cold, and pressure pain tolerance (55, 59, 60). Furthermore, when compared to Whites, Afro-American Blacks and other minority races tend to experience greater sensitivity to pain stimuli and less efficient descending pain inhibition evaluated through conditioned pain modulation or CPM (61–66).

## Irritable Bowel Syndrome

Irritable bowel syndrome (IBS) is a gastrointestinal disorder defined by chronic abdominal pain and alterations in bowel habits (67), that commonly co-occurs with other chronic pain conditions including headache and TMD (68). IBS is characterized by chronic visceral pain, which is poorly localized pain arising from the pelvic, thoracic, or abdominal organs (69), yet patients with IBS also suffer from abnormalities in gut-brain interactions (70). Patients with IBS display heightened pain sensitivity which may be attributed to a variety of mechanisms, including alterations in the processing of pain sensory information (both spinal and central), and afferent signaling of pain (71). As information surrounding endogenous pain modulation can be provided through QST paradigms, both pain facilitation and pain inhibition (72), quantifying sensory alterations in the IBS patient population offers a means whereby insight can be gained on pain modulatory systems.

Assessments of pain inhibition have most often been conducted in patients with IBS through the testing of conditioned pain modulation (CPM). Meta-analyses report patients with IBS display significantly diminished CPM in comparison with healthy controls, suggesting abnormalities with descending pain pathways (11, 12). Therefore, interventions which enhance deficiencies in pain inhibition, whether pharmacological, physiological, or electrical, may theoretically be helpful in the management of pain in patients with IBS (11). Importantly, this research also reports a high correlation between reduced CPM with psychological factors such as stress, anxiety, and pain catastrophizing, thus highlighting the complex relationship between mood, cognition, and CPM responses (12). Therefore, incorporating QST in the assessment of pain in patients with IBS, may lend insight on central vs. peripheral mechanisms and pain modulatory capabilities, which bears relevance when selecting therapeutic interventions.

## QST in Placebo/Nocebo Research

The placebo effect represents the neurobiological response to inert treatments and interventions (73). A large study evaluated differences in levels of placebo effects, based on expectations and prior experiences, between participants with chronic orofacial pain and healthy controls (74). The intervention consisted of painful thermal stimuli, along with a sham electrode the participants were told would decrease the pain produced by the device. Results showed similar levels of placebo effects, with more responders in the control group, whereas pain relief expectations were higher in the chronic pain group. Interestingly, the occurrence of placebo effects in both groups was not attributed to participant expectations, but rather to their prior experiences of having received the painful stimuli (74). In a study conducted to explore racial differences in pain and placebo effects, when compared to Whites, Afro-American Blacks demonstrated less efficient placebo hypoalgesia (57) and lower thermal heat tolerance. Importantly, there was a significant interaction with the race of the experimenters and the race of TMD participants hinting to potential healthy inequities and not just mechanisms of nociception. Moreover, QST varies in Afro-American Blacks but this variation is not linked to placebo effects (57).

The nocebo effect refers to worsening in symptoms when an inert substance or treatment is administered (73). While the relationship between nocebo and catastrophizing has been posited (75), Taub et al. (76) demonstrated the presence of pain catastrophizing in women with chronic low back pain. Performance of QST pre- and post-induction of pain catastrophizing, demonstrated increased pain in women with higher levels of pain catastrophizing, as well as extension of pain to areas where it was previously non-existent. Again, QST facilitated an objective measurement of pain in a controlled setting to study nocebo effects.

## QST in Special Populations/Alzheimer's Disease

Over 90% of individuals living with Alzheimer's disease (AD) are likely to experience pain at some point during the course of their dementia (77, 78), however, patient reported response of pain among individuals with AD has been inconsistent. A meta-analysis reports individuals with AD demonstrate higher pain sensitivity to experimental pain when using validated observational ratings of pain (79), and exhibit more pain behaviors when exposed to painful stimuli compared to individuals who are cognitively intact (80). Conversely, some studies note individuals with AD are less likely to report pain and use fewer analgesics compared with individuals without AD (81–85). Some individual items on observational instruments that measure pain are non-specific for pain exclusively and may be reflective of neuropsychiatric symptoms (restlessness, repetitive vocalizations) instead of pain. Several hypotheses have been proposed to explain differences in pain sensitivity and expression based on cognition. First, individuals with AD are unable to reliably report pain due to declining perceptual and communication abilities (86, 87). Second, individuals with AD experience fewer pain conditions, and third, these individuals experience alterations in nociceptive processing (e.g., peripheral sensory or central functioning) that impact their pain experience (84). The measurement of pain among individuals with AD may benefit from triangulation. The addition of QST measures to the assessment of pain among individuals with AD may provide neurophysiological data that complements the use of surveys and observational measures of pain.

To better understand whether altered nociceptive processing among individuals with AD contributes to altered pain sensitivity, some studies have utilized QST. Based on QST studies conducted in Italy and Australia, individuals with AD are reported to experience less pain sensitivity to temperature (88) and higher pressure pain thresholds (89) compared with controls. In contrast, groups in Denmark and the United States report diminished pain tolerance in individuals with AD compared with matched controls using QST (78, 80).

Use of QST among individuals with AD and related dementias has been limited. There are legitimate concerns regarding the reliability, safety, and ethical concerns of inducing discomfort in a vulnerable population. Additionally, challenges associated with obtaining informed consent in a population with impaired decision-making abilities contribute to its limited use. To best address such concerns, stimulus testing should be conducted over at least three trials, and false positive responses controlled for through application of a control or null stimulus (e.g. no filament or temperature applied). Safety strategies can also be put in place to minimize risks when working with older adults. Individuals with AD can be paired with a research team member during pain threshold testing who can assist with instructions and stimulus removal if the participant reports or exhibits signs of pain but does not withdraw. To help facilitate recruitment of individuals with AD, the proxy/legally authorized representative could be offered a trial experience of QST testing to better understand what the individual would experience.

## Discussion

QST has been used primarily in the research setting to characterize peripheral and central mechanisms underlying pain. Our illustration of QST's relevance across chronic pain conditions and within special populations, as investigated in our OASIS Center, supports its clinical utility to guide the selection of appropriate therapies and optimize patient outcomes. For instance, detection of peripheral sensory nerve function in LE chronic pain can lead to targeted mobility interventions, recognition of abnormal pain inhibitory pathways in patients with IBS can direct prescription of neuromodulator therapy, and assessment of altered nociceptive processing among individuals with AD can guide adequate pain control. Therefore, QST offers insight not only regarding physiological underpinnings of pain from a research perspective, its incorporation among our clinical assessment methods can potentially enhance the efficacy of our therapeutic approach, including non-pharmacological interventions.

Alongside the promise of QST in improving the characterization of pain, and in turn, the prescription of individualized treatment interventions, limitations of this methodology must also be addressed. As highlighted in our descriptions of QST in TMD and placebo research, sex and racial effects regarding pain sensitivity, and racial effects regarding placebo efficiency have been observed, and thus need be accounted for when analyzing results (57, 58). In addition, effects of age must be considered when incorporating QST, as loss of sensory function has been observed in older adults for cold, warmth, mechanical, and vibratory detection thresholds (90). Moreover, a recent systematic review with meta-analysis surrounding the use of QST in individuals with joint pain, reports an association between depression, pain catastrophizing, and physical activity level with several QST measures; therefore, such variables need be acknowledged when evaluating the relationship between pain and somatosensory function (91). Despite the potential of such cofounding factors, the ability of QST to detect and discriminate peripheral vs. central contributors to pain, and its potential to help guide targeted therapies, including non-pharmacological interventions, argues for its greater incorporation in research and clinical initiatives. Moving forward, efforts to shorten and standardize QST protocols across chronic pain conditions, and increasing portability of QST machines, will facilitate greater application of this methodology in research investigations, ease its adaptation to the clinical arena, and enhance our efforts toward reducing the burden of chronic pain.

## Author Contributions

KW, MG, NK, EG, AD, LC, and CR wrote sections of the manuscript. All authors contributed to the conception and design of the study, manuscript revision, read, and approved the submitted version.

## Funding

The authors would like to acknowledge funding and support from the National Institutes of Health, National Institute of Nursing Research, P30 Omics Associated with Self-Management Interventions for Symptoms (OASIS) Center (P30NRO16579 to BR, SD, and CR), and National Institute of Dental Craniofacial Research (1R01DE025946 to LC).

## Conflict of Interest

The authors declare that the research was conducted in the absence of any commercial or financial relationships that could be construed as a potential conflict of interest.

## Publisher's Note

All claims expressed in this article are solely those of the authors and do not necessarily represent those of their affiliated organizations, or those of the publisher, the editors and the reviewers. Any product that may be evaluated in this article, or claim that may be made by its manufacturer, is not guaranteed or endorsed by the publisher.
